# Experimental validation of computer-vision methods for the successful detection of endodontic treatment obturation and progression from noisy radiographs

**DOI:** 10.1007/s11282-023-00685-8

**Published:** 2023-04-25

**Authors:** Habib Al Hasan, Farhan Hasin Saad, Saif Ahmed, Nabeel Mohammed, Taseef Hasan Farook, James Dudley

**Affiliations:** 1 Department of Electrical and Computer Engineering, North South University, Dhaka, Bangladesh; 2 Adelaide Dental School, Faculty of Health and Medical Sciences, The University of Adelaide, Level 10, AHMS Building, Adelaide, South Australia 5000 Australia

**Keywords:** Malpractice, Endodontic failure, Obturation, Object detection, Deep learning

## Abstract

**Purpose:**

(1) To evaluate the effects of denoising and data balancing on deep learning to detect endodontic treatment outcomes from radiographs. (2) To develop and train a deep-learning model and classifier to predict obturation quality from radiomics.

**Methods:**

The study conformed to the STARD 2015 and MI-CLAIMS 2021 guidelines. 250 deidentified dental radiographs were collected and augmented to produce 2226 images. The dataset was classified according to endodontic treatment outcomes following a set of customized criteria. The dataset was denoised and balanced, and processed with YOLOv5s, YOLOv5x, and YOLOv7 models of real-time deep-learning computer vision. Diagnostic test parameters such as sensitivity (Sn), specificity (Sp), accuracy (Ac), precision, recall, mean average precision (mAP), and confidence were evaluated.

**Results:**

Overall accuracy for all the deep-learning models was above 85%. Imbalanced datasets with noise removal led to YOLOv5x’s prediction accuracy to drop to 72%, while balancing and noise removal led to all three models performing at over 95% accuracy. mAP saw an improvement from 52 to 92% following balancing and denoising.

**Conclusion:**

The current study of computer vision applied to radiomic datasets successfully classified endodontic treatment obturation and mishaps according to a custom progressive classification system and serves as a foundation to larger research on the subject matter.

## Introduction

Endodontic (root canal) treatment refers to the treatment sequence for the infected pulp chamber and root canals of a tooth in an effort to eliminate infection and protect the decontaminated tooth from future microbial invasion. Historically, root canal treatments have seen varying long-term success ranging from 31 to 96% based on strict criteria or from 60 to 100% based on loose criteria, with significant heterogeneity in the assessments of aggregated success rates [1].

Oral health requirements are not universally or equally met as geographic variables affect the quality of healthcare delivered and infrastructure provisioned for dental management. Similar to every other aspect of dentistry, professional experience and quality of equipment used dictates the level of success that can be achieved during endodontic practice. Therefore, it comes as no surprise that in inadequately provisioned dental healthcare systems there are frequent suboptimal endodontic treatments, with some leading to catastrophic failures. This is also paired with noise and artifact-prone radiomics that plagues older technology that is predominantly present in developing nations and rural practices owing to a lack of formal skill assessment [2]. There are numerous causes of root canal treatment failure, including but not limited to inadequate filling, poor filling, inappropriate filling, and the existence of methodological problems. A study of 100 cases evaluating possible causes of treatment failure showed that 46.9% of the root canals were underfilled while poorly filled and overfilled canals made up for 28.5% and 13% of the total cohort, respectively [3].

Outcomes of root canal treatment are determined by clinical symptoms presented by the patient such as pain and correlating the symptoms to post-treatment radiographs. The procedure is once again driven by experience, while an automated decision support system trained to identify suboptimal or endodontic mistreatment is still lacking. A model designed to identify and classify such outcomes can assist dental practitioners in validating their diagnoses and aid undergraduate students in training to become dentists. European judicial and legal institutions have lately introduced AI and its subsidiary applications in facial recognition and confirmation of criminal accusation can possibly find its way into dental litigations in the near future [4, 5]. Research on forensic applications of dental radiomics is already underway with Rabbani et. al [6] documenting the use of dentition data from panoramic radiograph to detect missing persons from disaster scenarios.

Machines mimicking human cognitive abilities are called artificial intelligence, or AI. Neural Networks (NN) are the building blocks of AI and are synthetic adaptive systems whose automated and unsupervised functionalities draw inspiration from the human brain's operations [7]. A neural network in computer vision, in this case the YOLO (You only look once) algorithm, predicts items within a picture in real-time and identifies them using ‘bounding boxes’ through object detection, which is a sophisticated and refined method of image classification. Thus, ‘object detection’ or ‘object recognition’ refers to the identification and location of items within an image that fall under one in a set of pre-established classes. To find objects and classes within images, YOLO employs Convolutional Neural Networks (CNN) which operates by obtaining an image, assign different weights to the objects within it, and then separate them from one another with remarkable speed. The current study developed a classification model trained using computer-vision algorithms YOLOv5s, YOLOv5x, and YOLOv7 from the YOLO family. YOLOv5s is smaller in size and faster to train while YOLOv5x requires more weighted parameters as minimum data to build a valid model, thus making it more reliable at the expense of longer processing times. Finally, YOLOv7, the latest version of its series aimed to secure a stable middle ground by increasing detection accuracy without decreasing the detection speed.

Prior to the introduction of deep-learning models for object detection, image processing-based algorithms were extensively utilized for image segmentation and detection in dentistry. However, both situations are prone to noise generation. An erratically transmitted signal's fluctuation produces noise and plagues images ranging from radiographs to low light photography and impede the AI’s ability to learn of a situation with maximum accuracy.

### Study rationale

Studies using machine learning and computer vision in endodontic treatment ranged from working length determination from radiographic images using artificial neural networks to the identification of canal morphology from 3D imaging such as cone-beam computed tomography [8]. However, most outcomes from radiographic images were based on successful endodontic canal obturations, with a study of computer-vision based deep learning to classify incomplete or failed endodontic canal obturation through radiomics still lacking. There are few studies published on computer vision and object detection in endodontic treatment. Researchers have proposed several image processing techniques and machine-learning algorithms for detecting dental decay from colored photographs and radiographic images. However, very few investigations have looked at object detection for endodontic treatment and none for suboptimal obturation. Finally, to the authors’ knowledge, no study has implemented computer vision to classify suboptimal and failed endodontic canal obturation, and potential endodontic malpractice.

Therefore, the aims of this study were to develop a novel in-house machine-learning classification system for endodontic obturation for implementation with computer-vision diagnostics to classify endodontic treatment outcomes from radiographic images. It was expected that the system would be able to classify outcomes accurately, irrespective of the noise and artifacts present within the original radiograph.

### Objectives

1. To evaluate the impact of artifact noise and dataset imbalance and subsequent augmentation on computer-vision models when classifying endodontic treatment outcomes
2. To develop an object detection model to accurately predict obturation outcomes and suboptimal endodontic treatment from radiographic images

## Materials & methods

### Reporting protocols

The current in vitro retrospective study was conducted and reported in accordance with the Standards for Reporting Diagnostic accuracy studies (STARD) 2015 guidelines [9] and Minimum Information about Clinical Artificial Intelligence Modeling (MI-CLAMS) 2021 protocol [10].

### Ethics

The study was deemed ‘negligible risk’ according to the relevant ethics committees and was therefore exempt from ethical review.

### Study tools

All radiographic images were provided for deep learning as JPEG files at maximum quality. Virtual areas of interest (v-ROI) were identified through “bounding boxes” (creating boxes around ROIs inside photographs) on an open-source Python-based image labeling system and labeled by the two dentists allowing revision until complete in-person agreement (κ = 1.00) was attained regarding placement of bounding boxes. Virtual labeling was carried out inside the LabelImg.py system for computer-vision object detection (YOLO; Bochkovskiy et al. 2020).

### Participant characteristics

250 deidentified digital radiographic images of endodontic obturation and failed endodontic canal obturation were obtained via anonymised submissions from dental practitioners. The eligibility criteria included submission of deidentified patient radiographs in physical or printed copies (either periapical, bitewing, or panoramic) that demonstrated one of the four target conditions (described in next subsection) according to the submitting practitioner’s judgment. The anonymized submission requested inclusion of radiographs of endodontic treatment performed on patients visiting the dental practices from remote rural communities without resolution of initial symptoms or referred patients who were incorrectly diagnosed or treated by dental ‘quacks’, as confirmed by the practitioners who followed up on the matter with the regulating Dental Board. Quacks are individuals who do not hold a formal dental degree but illegally perform complex dental procedures in poverty-stricken communities without regulation. [11] It was requested that radiographs of the teeth subsequently obturated by the submitting practitioner or retreated following best practice protocols in the last one month be also supplied. This was to help the computer-vision models to learn and differentiate obturation levels on the same environment and tooth morphology. Exclusion criteria included radiographs following endodontic or periodontal surgery or images that demonstrated surgical fixation units such as implants, screws, and miniplates. Systemic conditions or medical records were neither collected nor considered during image exclusion. All resultant images were screened and annotated by two dentists for acceptability of selection criteria and images that did not generate κ = 1.00 interrater agreement were discarded, leading to 240 images that were fully agreed upon. The images contained treatment outcomes ranging from complete treatment to suboptimal obturation and was noted that the dataset was imbalanced. This was addressed and discussed in the following sections.

### Target condition and classification system

An in-house classification system was designed to classify endodontic obturation progression for deep learning from radiographic images in the following capacity:

Class 1: no endodontic treatment performed

- No canal sealed irrespective of carious lesion or periapical radiolucency present
- Possible pulp chamber or canal exposure without dental intervention
- Dental intervention performed with vital tooth crown or conservative restoration

Class 2: incomplete endodontic obturation performed

- Radiographs of canals obturated more than 1/3^rd^ of the canal length but not up to the apical constriction
- Radiologically evident missed canals following obturation
- incomplete obturation thickness with visible canal radiolucency adjacent to the obturation material
- Radiographs taken of teeth showcasing root canals during mid-endodontic treatment
- Placement of posts into canals without adequate canal obturation underneath

Class 3^1^: suboptimal endodontic treatment

- Radiographs of canals obturated to less than 1/3^rd^ of the canal length with or without evidence of an iatrogenic mishap such as ledge formation or proximal stripping
- Evident pulp chamber or canal perforation with subsequent obturation with or without perforation repair
- Placement of permanent prostheses like crowns or fixed partial dentures over endodontically treated teeth without adequate obturation
- Endodontic treatment of unrestorable teeth possessing roots with less than 1/3^rd^ of periodontal attachment and bone support

Class 4: complete endodontic obturation performed

- Complete canal obturation irrespective of periapical radiolucency
- Complete canal obturation unaffected by loss of periodontal attachment, furcation involvement or bone loss

### Data pre-processing

The radiographs were first converted from RGB to grayscale to reduce information-per-image and facilitate faster processing and lower storage requirements [12]. Higher resolution images would greatly reduce processing time and therefore scaled down to a standard 416 × 416 pixels for optimum consistency in AI training rates [13]. The original radiographic images contained noise, and therefore, 3 versions of the current dataset were created for comparative evaluation. Afterwards, 7 different augmentation methods were applied on the training sets using a python-based augmenter that included various degrees of rotation, vertical and horizontal flipping, inverting, and blurring.

**Fig. 1:**
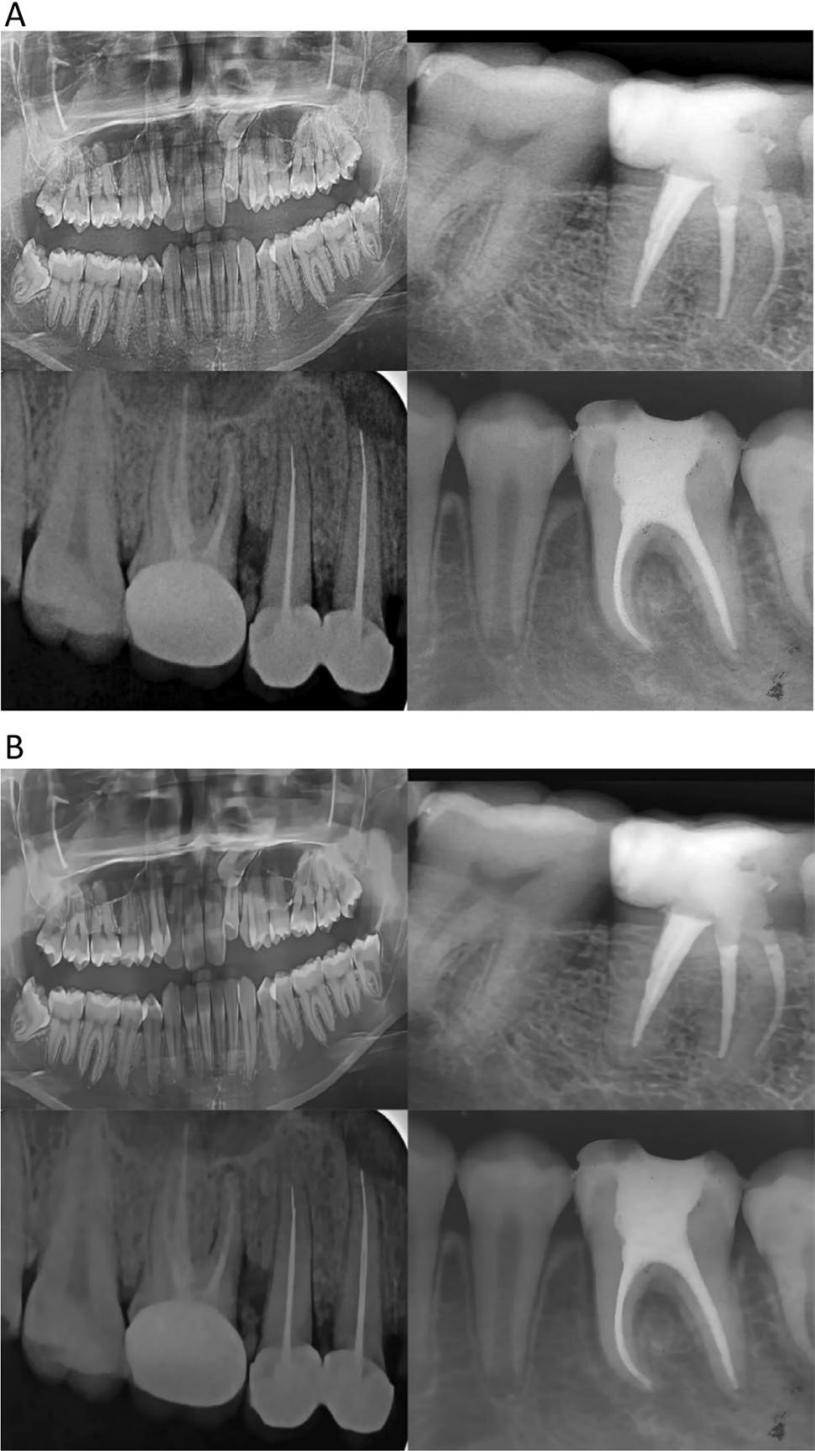
Comparison between **A** noised and **B** denoised image

**Fig. 2:**
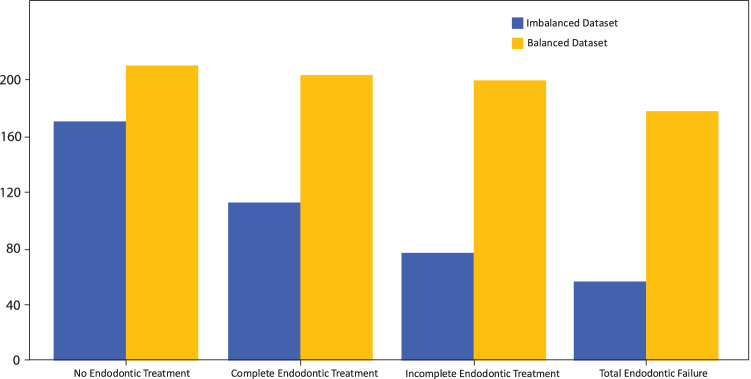
distribution of dataset before and after balanced augmentation

1. *Imbalanced dataset with noise (original dataset)*. The original dataset had 240 images that were split as 75% (180 images) for the training set, 5% (12 images) for the validation set, and 20% (48 images) for the test set. This split was performed in all 3 versions. The training dataset was augmented seven folds using data augmentation methods resulting in 1260 images.
2. *Imbalanced dataset after denoising*: A denoising autoencoder is pre-trained to receive noisy image inputs and attempts to predict what the denoised state would appear as. A “noise” within an image is commonly defined as a random variance of radiance or color features in images, which is frequently created by the technological limitations of the image collection sensor or by adverse environmental conditions. Denoising autoencoders creates distorted versions of the input images by adding random noise and then attempts to restore the distorted image to original input. Such a model performed poorly on the present dataset and therefore a BM3D (Block Matching 3D) denoising algorithm was used in its stead that was recently proven to have better noise removal capabilities without affecting image quality [14, 15], Fig. 1 shows a comparison before and after the denoising technique applied.
3. *Balanced dataset after denoising*: The current dataset lacked uniform distribution and was moderately skewed. To address this, minority classes were identified followed by selective augmentation [16] resulting in a combined balanced dataset of 424 images. The role of selective augmentation has been shown in Fig. 2.

### Model description

This section discusses the deep-learning model applied for endodontic treatment detection. The YOLO algorithm identifies objects in real-time within photographs. Each image receives an S x S grid with each grid predicting N bounding boxes and confidence [17]. The bounding box's accuracy and whether it genuinely includes an object are reflected in the confidence parameter (regardless of class). Additionally, YOLO predicts the classification score for each box and each training class. Convolutional neural networks (CNN) are then used by the YOLO algorithm for instant recognition and require only one forward propagation. This means that a single algorithm, once run, can perform prediction models throughout the entire image [18]. In the current investigation, to improve detection accuracy, YOLOv5 and YOLOv7 models were pre-trained on the MS COCO dataset. This method or pre-training a model on a separate dataset prior to the actual learning is defined as ‘transfer learning’ and can greatly reduce training time and logistic requirements.

**Fig. 3:**
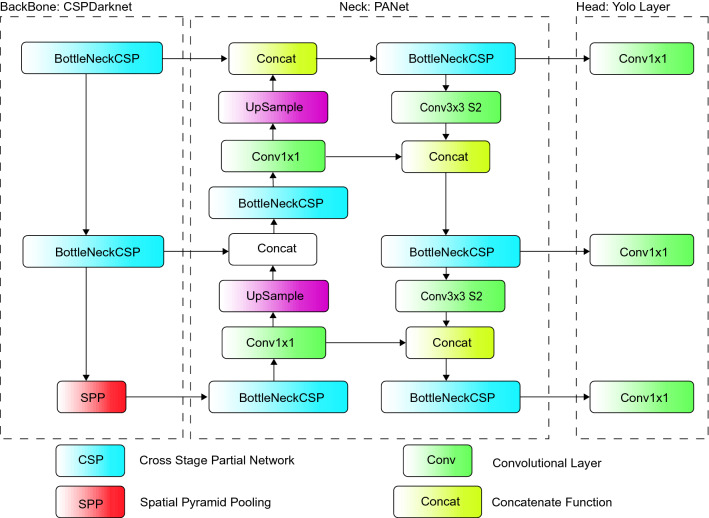
YOLOv5 architecture applied within the current study

1. YOLOv5: YOLOv5 had several pre-trained models with differences in size, layer, and inference time of which YOLOv5s is smaller and computationally less demanding and YOLOv5x is extensive and highly accurate. Hence these models were chosen, and are described in Table 1. The entire architecture of YOLOv5 is shown in Fig. 3 and is built on 3 architectural blocks: Backbone, Neck, and Head [19]. The Backbone, in this model being CSPDarknet (Cross Stage Partial Network), extracts important features from an input image. The neck, here YOLOv5 PANet, is used for the features pyramid. A ‘Feature Pyramid Network’ is a feature extractor algorithm that produces proportionately scaled-up convolutional feature maps on several layers from a single-scale picture of any size as its input. This helps to resize and scale the same object and proceeds to modeling the object on unseen data. The head is used for output detection results, namely class, score, localization, and size.

**Table 1:** YOLO models implemented and their property characterization

Model	Size	Layer	Inference Time
YOLOv5s	14mb	213	17 ms
YOLOv5X	168mb	444	49 ms
YOLOv7	72mb	306	28 ms

An artificial neural network can learn complicated patterns in the data with the aid of an activation function, which is an algorithm that was introduced to the current neural network. The activation process selects the signals to be sent to the following neuron. YOLOv5 in the current study used ‘LeakyReLU’ and ‘Sigmoid’ as activation function options [5]. Optimisers are programs or techniques that modify the neural network's properties, such as its weights and learning rate, to minimize ‘loss’. Here, YOLOv5 used SGD [24] and ADAM [28] as their optimisers options [19].

The loss function is commonly used in the object detection to clarify the degree of change between the predicted and actual values of the model and is of particular importance in the present investigation as the model needed to correctly classify suboptimal treatment to endodontic malpractice. The loss function in YOLOv5 used binary cross-entropy with logit loss that included three parts: bounding box regression loss, confidence loss, and classification loss [20].

$$L_{GIoU} = \mathop \sum \limits_{i = 0}^{{S^{2} }} \,\sum\limits_{J = 0}^{B} {I_{i,j}^{obj} } \left[ {1 - IoU + \frac{{A^{c} - U}}{{A^{c} }}} \right]$$

where ${S}^{2}$ represents the number of grids in an image and B represents the number of bounding boxes in each grid. When an object exists in a bounding box, ${I}_{i,j}^{obj}$ is equal to 1, otherwise it is 0.[20].

Confidence Loss:

$$L_{comf} = - \mathop \sum \limits_{i = 0}^{{S^{2} }} \,\sum\limits_{J = 0}^{B} {I_{i,j}^{obj} } \,\left[ {\hat{C}_{i}^{j} log\left( {C_{i}^{j} } \right) + \left( {1 - \hat{C}_{i}^{j} } \right)log\left( {1 - C_{i}^{j} } \right)} \right] - \lambda_{noobj} \mathop \sum \limits_{i = 0}^{{S^{2} }} \,\sum\limits_{J = 0}^{B} {I_{i,j}^{noobj} } \left[ {C_{i}^{j} log\left( {C_{i}^{j} } \right) + \left( {1 - C_{i}^{j} } \right)log\left( {1 - \hat{C}_{i}^{j} } \right)} \right]$$

Classification Loss:

$$L_{class} = - \sum\limits_{i = 0}^{{S^{2} }} {I_{i,j}^{noobj} } \sum\limits_{ceclasses} {[\hat{P}_{i}^{j} \left( c \right)log\left( {I_{i}^{j} \left( c \right)} \right) + \left( {1 - \hat{P}_{i}^{j} \left( c \right)} \right)log\left( {1 - P_{i}^{j} \left( c \right)} \right)}$$

where ${\widehat{P}}_{i}^{j}(c)$ represents the probability of predicting the endodontic object as class c, and ${P}_{i}^{j}(c)$ represents the probability of the object actually belonging to class c.

The total loss function can be represented as:

$$LOSS = L_{GIoU} + L_{conf} + L_{class}$$

**Fig. 4:**
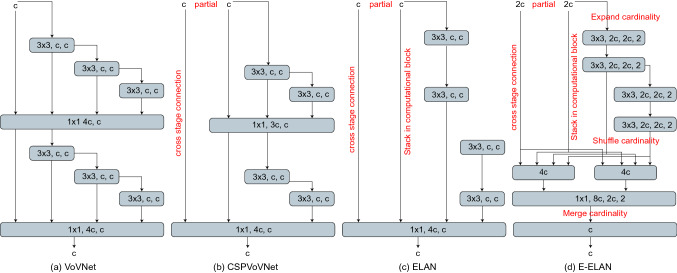
YOLO v7 architecture applied within the current study

2. YOLOv7: While very limited research has been done on the model in healthcare, YOLOv7 sports faster training times and better diagnostics thus rendering it capable of detecting small objects and changes. The entire architecture of YOLOv7 [14] is shown in Fig. 4. The computational building component of the YOLOv7 backbone is called E-ELAN (Extended Efficient Layer Aggregation). It draws influence from earlier studies on network effectiveness. It was created by looking at the following elements that affect speed and accuracy: Cost of memory access, I/O channel ratio, operation in elements, activations, and gradient path. The model description is shown in Table 1.

### Evaluation metrics

**Fig. 5:**
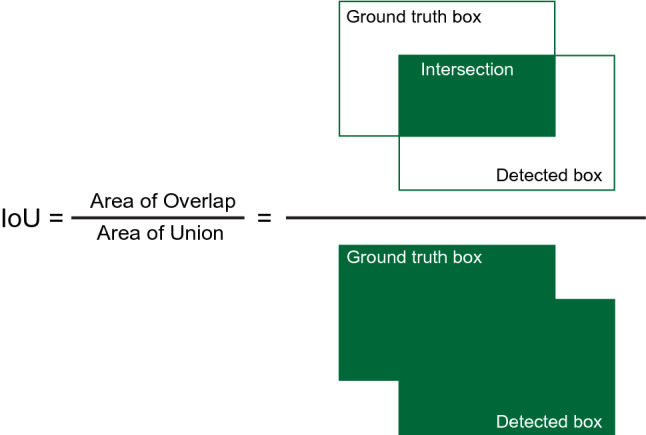
Intersection over union model

The performance of classification or object detection models were evaluated using a variety of metrics, including precision, recall, average precision, specificity, sensitivity, and F1 score. To evaluate the performance of the developed model, three evaluation metrics were considered: mean average precision (mAP), precision, and recall. The accuracy of a model to detect objects is measured by the mAP [21], which is used as the primary evaluation metric for an object detection model. The performance of the model improves with increasing mAP values. mAP is simply determined by the mean average of the average precision (AP) of each class based on a predetermined IoU (intersection-over-union) threshold. The IoU (Fig. 5) measures the overlapping area between the expected bounding box (B_p_)and the ground truth bounding box (B_gt_) [21]. The formula of IoU is

$$IoU = \frac{{area\left( {B_{p} \cap B_{gt} } \right)}}{{area\left( {B_{p} \cup B_{gt} } \right)}}$$

In the current investigation, YOLOv5 and YOLOv7 were measured for average precision with a default IoU value set to 0.5. Here, precision was used to measure how accurately the model could produce positive predictions.

$$Precision = \frac{TP}{{TP + FP}}$$

$$Recall = \frac{TP}{{TP + FN}}$$

$$mAP^{\prime}\, = \,\frac{1}{N}\,\sum\limits_{i = 1}^{N} {AP_{i} }$$

Here,

- APi is the Average precision in the ith class and N is the total number of classes
- TP (True Positive): Actual class is positive and predicted positive
- FP (False Positive): Actual class is negative but predicted positive
- FN (False Negative): Actual class is positive but predicted negative.

Based on the IoU threshold, the YOLO model distinguishes between true positives (TP) and false positives (FP). In the current model, when the IoU threshold was greater than 0.5, it was regarded as a positive class, and when it was lower, it was regarded as a false positive class.

The hardware and software parameter of all experiments in this section are as follows:

This computational modeling was carried out within Collaboratory (Google Inc.) using Google’s Cloud Platform. The system ran on Pytorch v1.12.1 framework, CUDA v11.2, and was powered by a Tesla T4 Graphics processing unit. The algorithms were coded using Python 3.7.13 according to the PEP 8 guidelines.

A parameter whose value is utilized to regulate the learning process is known as a hyperparameter. The hyperparameter values we kept constant across the entire learning process with a Stochastic Gradient Descent used as an optimizer [22]. The learning rate was 0.01. Batch size was 16. Image size was 416 × 416. All experiments were run on 100 epochs, i.e., the number of cycles/passes that the machine-learning algorithm made across the full training dataset.

## Results

**Fig. 6:**
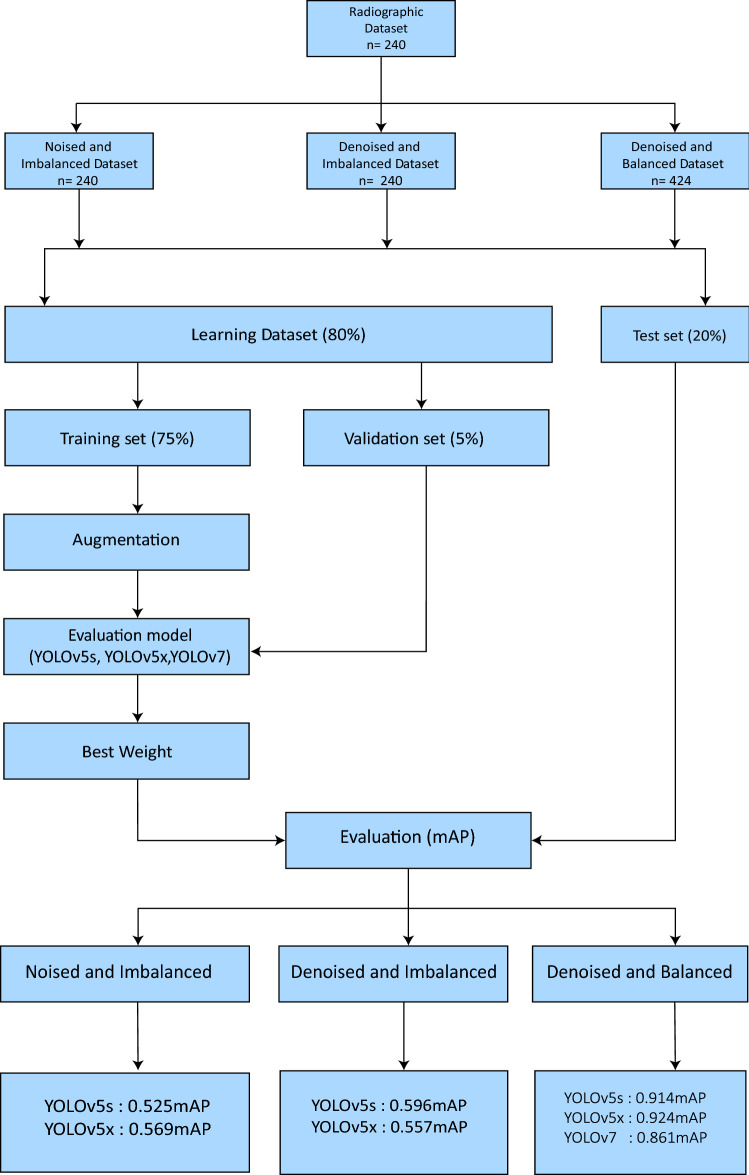
STARD flowchart summary

Object detection models YOLOv5s, YOLOv5x, and YOLOv7 were employed and trained on three different versions of datasets: ‘noisy and imbalanced’, ‘denoised and imbalanced’, and ‘denoised and balanced’. Figure 6 summarizes the workflow employed to achieve the desired deep-learning outcome. Additionally, the pre-trained model’s configuration file was changed so that it corresponds to the four classes this study has and their names. All the results were gathered after validating the trained models with their corresponding test (unseen) dataset. The models were then trained for 100 epochs in each experiment.

The original dataset was imbalanced and noisy (Table 2). Therefore, the images underwent soft augmentation prior to training as the original 240 images were impractical in generating appreciable results. This led to 1260 images. The overall accuracy for the YOLOv5s and YOLOv5x model was 86.4% and 89.1% respectively. Where the individual class ‘*No Endodontic Treatment’* had 100% accuracy for both models. However, there were some variations among the root canal classes in terms of the models. YOLOv5s and YOLOv5x models' accuracy for ‘*Completed Endodontic Treatment’* class was 80% and 87%, respectively with a 7% prediction improvement with YOLOv5x. However, YOLOv5s and YOLOv5x, exhibited accuracy rates of 68% and 83%, respectively for *‘Incomplete Endodontic Treatmen*t’. False positive rates for ‘*Incomplete Endodontic Treatment*’ class were substantially higher for both models (73% for YOLOv5s and 40% for YOLOv5x) with minimal false positives on the other classes. Of note, the testing accuracy for ‘*Total Endodontic Failure’* class was 86% in YOLOv5s and 89% in YOLOv5x.

**Table 2:** Test accuracy features of the Imbalanced dataset with noise

Models	Classes	TP	TN	FP	FN	F1-Score	Specificity	Sensitivity	Accuracy	Total accuracy
YOLOv5s	No endodontic treatment	0.42	2.3	0	0	1	1	1	1	0.864
	Complete endodontic treatment	0.77	1.64	0.08	0.53	0.72	0.95	0.59	0.8	
	Incomplete endodontic treatment	0.2	1.86	0.73	0.23	0.29	0.72	0.47	0.68	
	Total endodontic failure	0.64	1.97	0.18	0.23	0.76	0.92	0.74	0.86	
YOLOv5x	No endodontic treatment performed	0.42	2.24	0	0	1	1	1	1	0.891
	Complete endodontic treatment	0.82	1.5	0.05	0.29	0.83	0.97	0.74	0.87	
	Incomplete endodontic treatment	0.33	1.88	0.4	0.05	0.59	0.82	0.87	0.83	
	Total endodontic failure	0.55	1.82	0.09	0.2	0.79	0.95	0.73	0.89	

Table 3 consists of values that were collected after the denoising step. It demonstrates the increase in accuracy for both models across all classes. But because of the dataset’s skewness, some classes underperformed as expected. The overall accuracy for the YOLOv5s and YOLOv5x model was 90% and 72% respectively. The overall accuracy of the YOLOv5s model increased by 3.6% for all four classes on the denoised and unbalanced dataset. Additionally, the False Positive Rate for the class of incomplete endodontic treatment was reduced from 73 to 20% while increasing the diagnostic accuracy from 68 to 83%. Additionally, the accuracy rose by 6% for the ‘Completed Endodontic Treatment’ Class while remaining unchanged for the ‘No Endodontic Treatment’ Class. ‘Total Endodontic Failure’ class accuracy was marginally improved by 4%. The technique of denoising positively affected YOLOv5s but decreased accuracy for YOLOv5x. This shows the denoising approach had a significant impact on the overall model performance. Compared to the Noised Imbalanced Results it increased the accuracy for YOLOv5s model, but the accuracy dropped significantly for the YOLOv5X model.

**Table 3:** Test accuracy feature of the Imbalanced dataset after denoising

Models	Classes	TP	TN	FP	FN	F1-Score	Specificity	Sensitivity	Accuracy	Total accuracy
YOLOv5s	No endodontic treatment	0.31	2.51	0	0	1	1	1	1	0.904
	Complete endodontic treatment	0.82	1.61	0.1	0.29	0.81	0.94	0.74	0.86	
	Incomplete endodontic treatment	0.67	1.67	0.2	0.28	0.74	0.89	0.71	0.83	
	Total endodontic failure	0.45	2.1	0.27	0	0.77	0.89	1	0.9	
YOLOv5x	No endodontic treatment performed	0.38	2.36	0.88	0.03	0.41	0.73	0.93	0.75	0.721
	Complete endodontic treatment	0.77	2.41	0.18	0.29	0.77	0.93	0.73	0.87	
	Incomplete endodontic treatment	0.47	2.62	0.33	0.23	0.63	0.89	0.67	0.85	
	Total endodontic failure	0.55	2.08	0.09	0.93	0.52	0.96	0.37	0.72	

The initial dataset had 240 images. Selective augmentation of the minority class images (i.e., the classes with lesser data) produced 424 images. The modified dataset was then split into three sets of data not previously introduced during the selective augmentation, which produced 2226 images. This was achieved after performing 7 forms of augmentation on the training datasets. The results have been demonstrated in Table 4. Both the YOLOv5s and YOLOv5x models benefitted from heightened accuracy with the denoised balanced dataset, at 98.9% and 98.4%, respectively. YOLOv7 in comparison produced an accuracy of 95.4%. False positive rates were < 1% for each class within the three models with better precision and specificity. The outcomes were comparable and had diagnostic accuracies ranging from 95 to 99% for each class.

**Table 4:** Test feature characteristics of the balanced dataset after denoising

Models	Classes	TP	TN	FP	FN	F1-Score	Specificity	Sensitivity	Accuracy	Total accuracy
YOLOv5s	No endodontic treatment	0.65	2.93	0.04	0.02	0.96	0.99	0.97	0.98	0.989
	Complete endodontic treatment	0.95	2.6	0.03	0.06	0.95	0.99	0.94	0.98	
	Incomplete endodontic treatment	0.96	2.61	0.04	0.03	0.96	0.98	0.97	0.98	
	Total endodontic failure	0.95	2.65	0.02	0.02	0.98	0.98	0.98	0.99	
YOLOv5x	No endodontic treatment performed	0.75	2.94	0.04	0.02	0.96	0.99	0.97	0.98	0.984
	Complete endodontic treatment	0.97	2.7	0	0.08	0.96	1	0.92	0.98	
	Incomplete endodontic treatment	0.94	2.73	0.06	0.02	0.96	0.98	0.98	0.98	
	Total endodontic failure	0.95	2.74	0.04	0.02	0.97	0.99	0.98	0.98	
YOLOv7	No endodontic treatment performed	0.69	2.74	0.02	0.05	0.95	0.99	0.93	0.98	0.954
	Complete endodontic treatment	0.85	2.55	0.08	0.02	0.94	0.97	0.98	0.97	
	Incomplete endodontic treatment	0.88	2.43	0.08	0.11	0.9	0.97	0.89	0.95	
	Total endodontic failure	0.82	2.52	0.08	0.08	0.91	0.97	0.91	0.95	

The mean average precision (mAP) comparison for all tested models across the three dataset versions is shown in Table 5. The length of time required for forward propagation is referred to as the inference time. The inference time was divided by one to get the number of frames per second. The noised and unbalanced dataset's mAP for YOLOv5s and YOLOv5x was documented at 0.525 and 0.569, respectively. This was incrementally improved to 0.596 and 0.557 respectively for denoised and unbalanced and finally produced 0.914 and 0.924 for denoised and balanced datasets, respectively. The mAP for YOLOv7 was limited to 0.861. In addition, YOLOv5s models produced very fast inference times of 12–17 ms, but the YOLOv5x models had substantially slower inference times of 49–60 ms.

**Table 5:** Mean average precision for noise correction and balancing of dataset

Dataset	Model	Precision	Recall	mAp	Inference
Noised & imbalanced	YOLOv5s	0.597	0.534	0.525	12 ms
	YOLOv5x	0.655	0.539	0.569	58 ms
Denoised & imbalanced	YOLOv5s	0.561	0.602	0.596	12 ms
	YOLOv5x	0.599	0.528	0.557	59 ms
Denoised & balanced	YOLOv5s	0.945	0.845	0.914	17 ms
	YOLOv5x	0.954	0.878	0.924	49 ms
	YOLOv7	0.822	0.803	0.861	28 ms

**Fig. 7:**
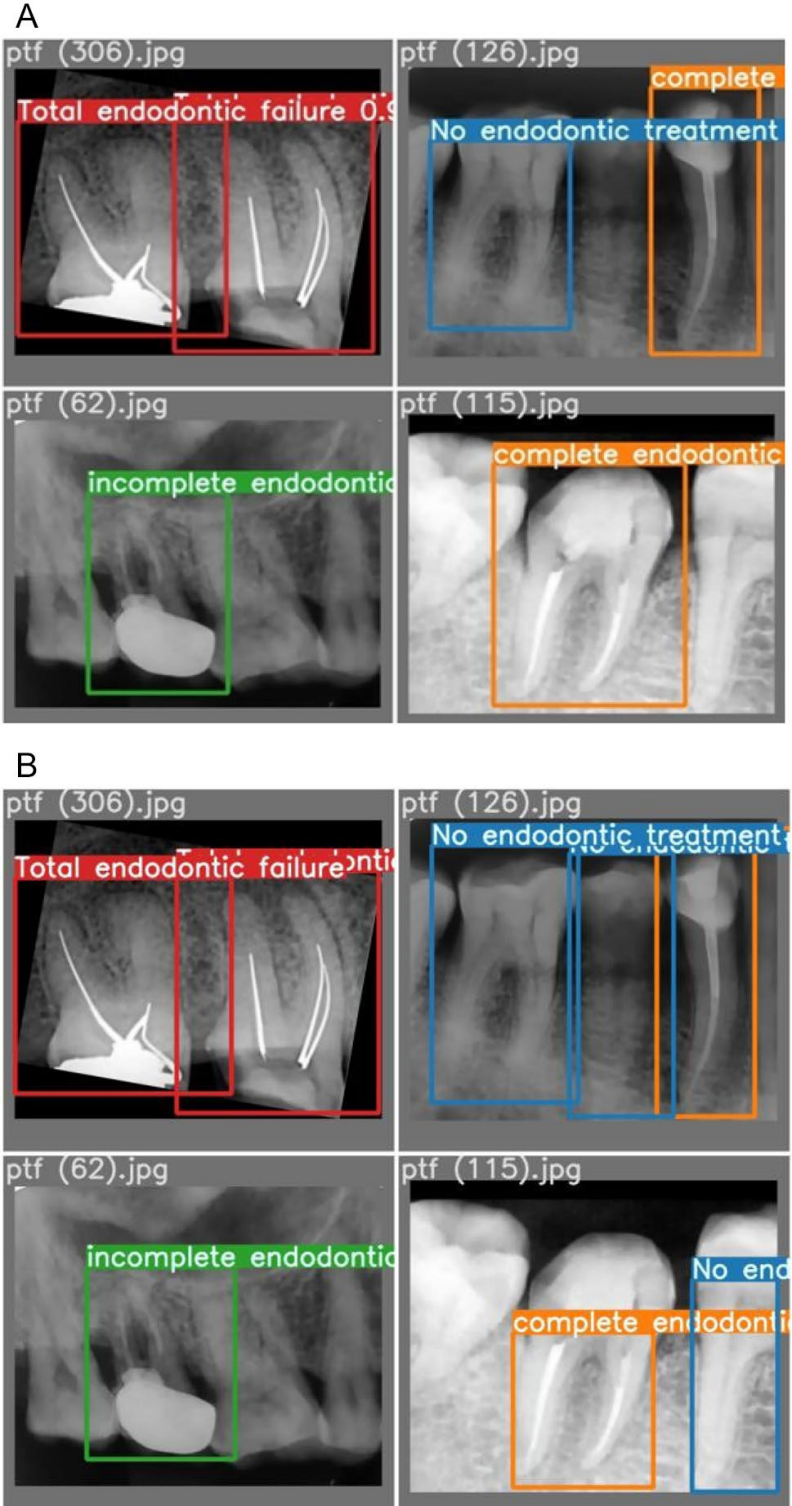
Comparison of predicted label **a** vs actual labels **b** from test data

**Fig. 8:**
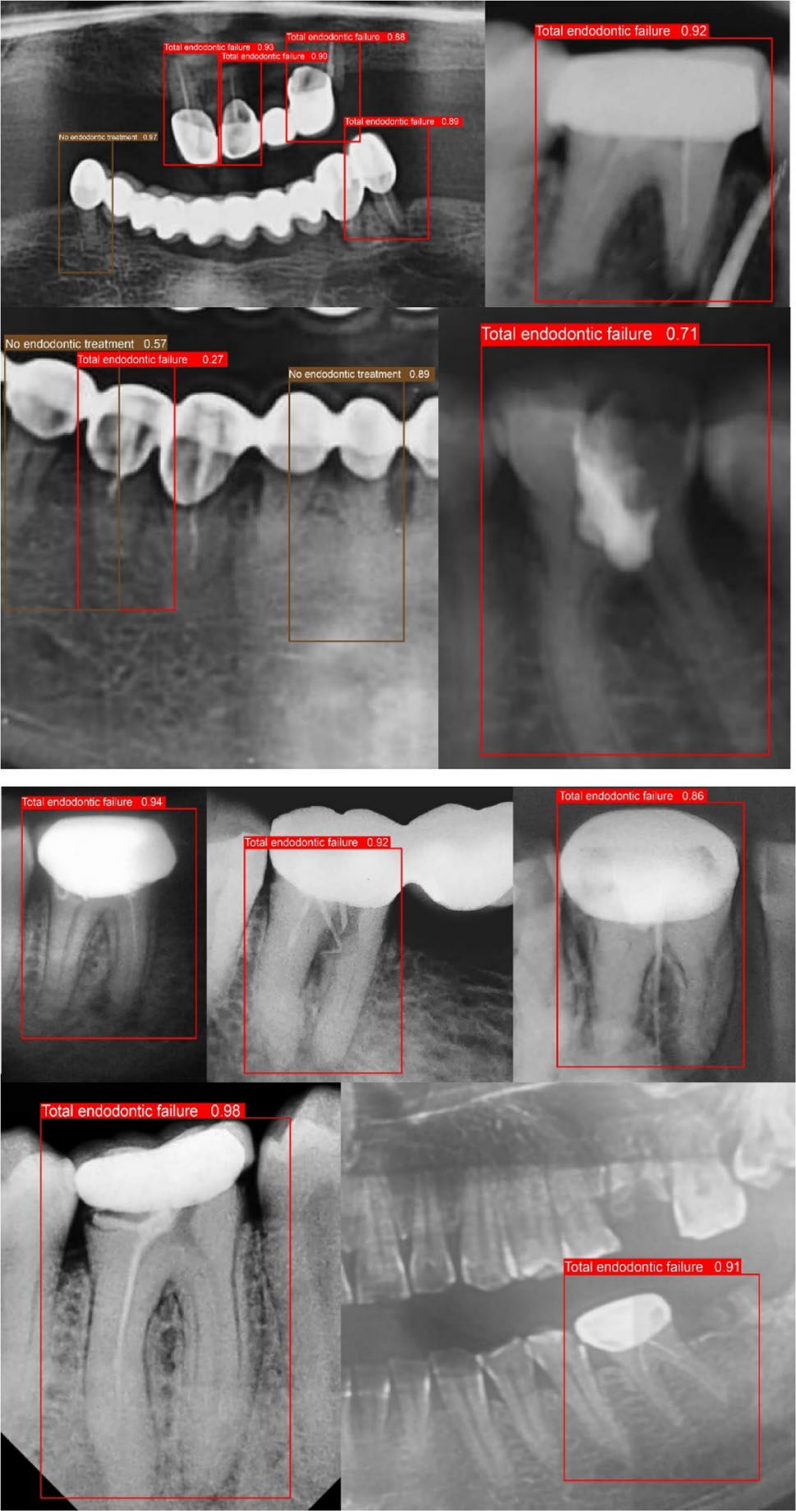
Prediction accuracy across the dataset of total endodontic failures and suboptimal treatment outcomes

**Fig. 9:**
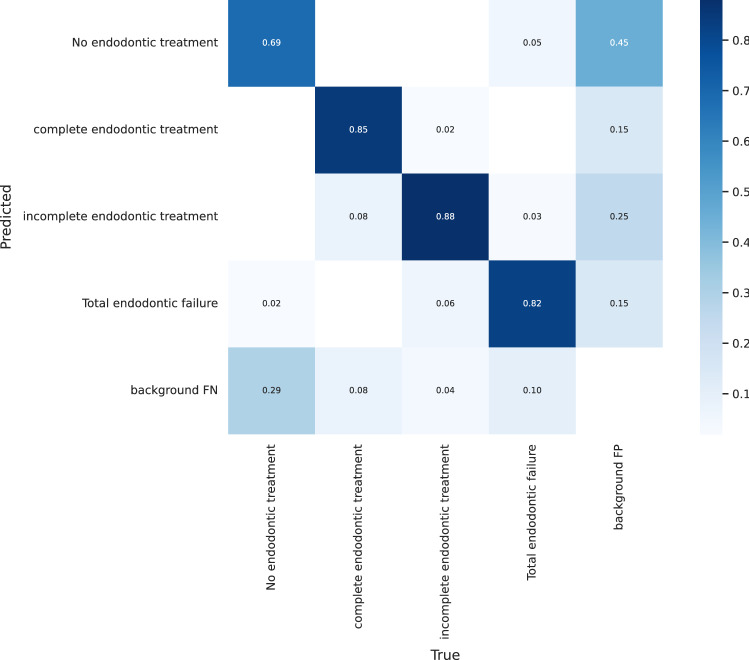
Confusion matrix of YOLOv7. (*Actual values are shown along the X-axis, and”Predicted” values are shown along the Y-axis.*)

Figure 7 shows a side-by-side comparison of the models’ results on the test set images. Actual labels are shown in image (a), while the labels predicted by the YOLOv7 model are shown in the image (b). The mAP following denoising and imbalance correction have been documented in Table 5. Of note, Fig. 8 highlights some of the predictions made on the ‘Total Endodontic Failure’ class. Figure 9 shows the confusion matrix of the currently trained YOLOv7 model on the denoised and balanced dataset. This is trained using YOLOv7 and relies on a denoised balanced dataset.

**Fig. 10:**
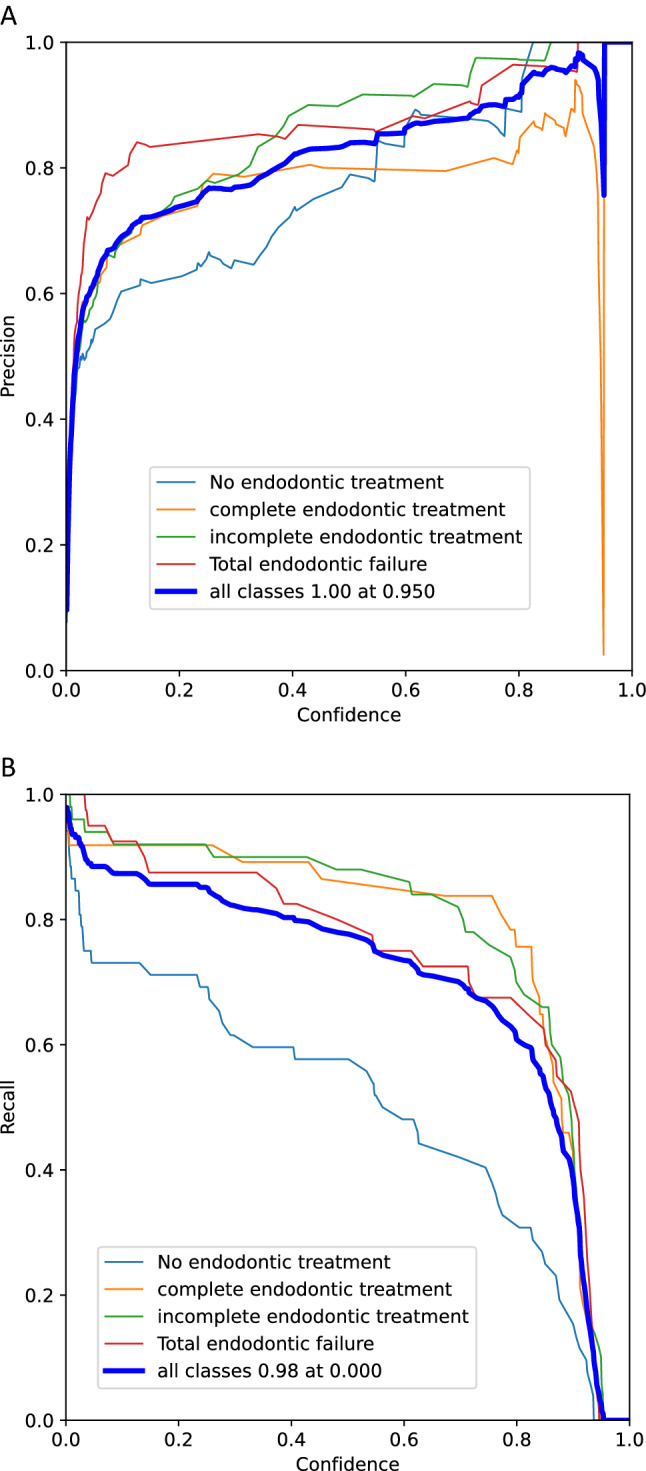
visual representation of test outcomes as **A)** Confidence vs precision and **B)** Confidence vs recall graphs

**Fig. 11:**
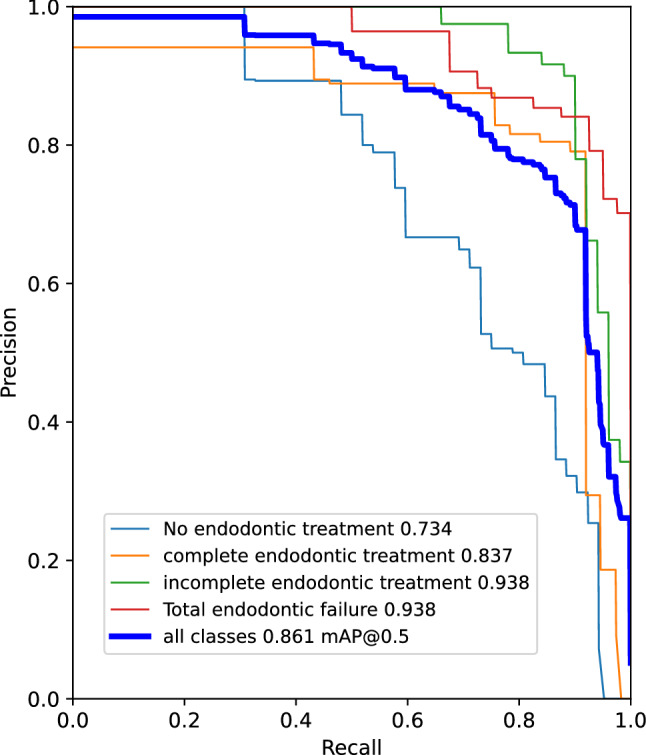
Precision recall graph summarizing the trade-off between true positive rate and positive predictive values

To eliminate false positives and guarantee that a projected bounding box has a specific minimum score, a confidence score threshold was set [17]. Fig. 10 depicts the confidence vs. precision graph, which slopes upward. This demonstrated that the average precisions improved relative to confidence while the recall curve had a negative slope against confidence. (Fig. 10) Finally, a Precision-Recall graph was formulated (Fig. 11) that summarized the trade-off between the model’s true positive rate and positive predictive value when different probability thresholds were used.

## Discussion

The current study developed a computer-vision directed classification system for endodontic obturation progression following denoising and balancing of radiomic dataset. To the authors’ knowledge, no previous study applied such a progressive classifier for endodontic obturation radiomics. In clinical endodontics, radiographs are often repeated if the images are blurry and not of an acceptable diagnostic standard [23]. Furthermore, when repeats are not indicated, a lack of practitioner experience or inadequate time spent with a radiograph may lead to inaccurate interpretations of endodontically obturated teeth and the possible need for retreatment. The issue becomes exponentially worse when the incident occurs in understaffed public practices within developing countries where practitioners have to diagnose hundreds of radiographs per day. The general fatigue and time constraints, among many other reasons, might lead to under-reporting of some incidental findings, or worse, accidental omission of critical information about a tooth to be treated [24]. The current report applied an automated clinical decision support system based on real-time computer-vision architectures that can diagnose images of radiographs in milliseconds, highlighting all areas of interest in the radiograph for the practitioner to view and take appropriate action upon. Such a method implemented through smartphone applications or smart glasses becomes especially useful in public hospitals and rural clinics of developing countries where traditional, blurry, blue-tinted, error-prone radiographs are still being viewed over a lightbox [23, 24]. Further iterations of such an implementation can also help licensed practitioners quickly screen the quality of work performed by quacks. who are known to administer questionable treatment to patients in poorer communities across developing country [11].

Previous reports of automated prediction of failed endodontic obturation were documented from patient history and symptoms upon follow-ups using logistic regression (logR), random forests (RF), gradient boosting machine (GBM), and extreme gradient boosting (XGB) for machine-learning driven predictive modeling [25]. Herbst’s study digressed from the current investigation in that the investigators did not approach the problem from a deep-learning perspective and obturation failure could only be partially predicted. The current study trained the radiomic dataset in several layers, first teaching the model to detect endodontic canal obturation, followed by categorizing the quality of obturation.

Advances in deep-learning frameworks include a system called DENTECT that was designed to recognize five dental treatment procedures including endodontic obturation and periapical lesion therapy, and concurrently numbered the dentition on panoramic radiographs using the FDI notation [26]. DENTECT was trained on 1005 photographs and followed expert annotations, whereas the current model was trained and validated with over 2000 augmented images. While monitoring periapical treatment is appealing, a periapical radiolucency was not classified as a failure within the current study as the lesion resolutions vary wildly, can take between 3 and 6 months to commence reduction in size, and deep-learned radiomic driven lesion therapy may not be clinically reliable. Furthermore, a previous systematic review found that ‘expert’ annotations were largely dependent on the years of experience held by the practitioner, with new professionals faring worse than the models trained by practitioners with 5 to 20 years of clinical experience [27].

**Fig. 12:**
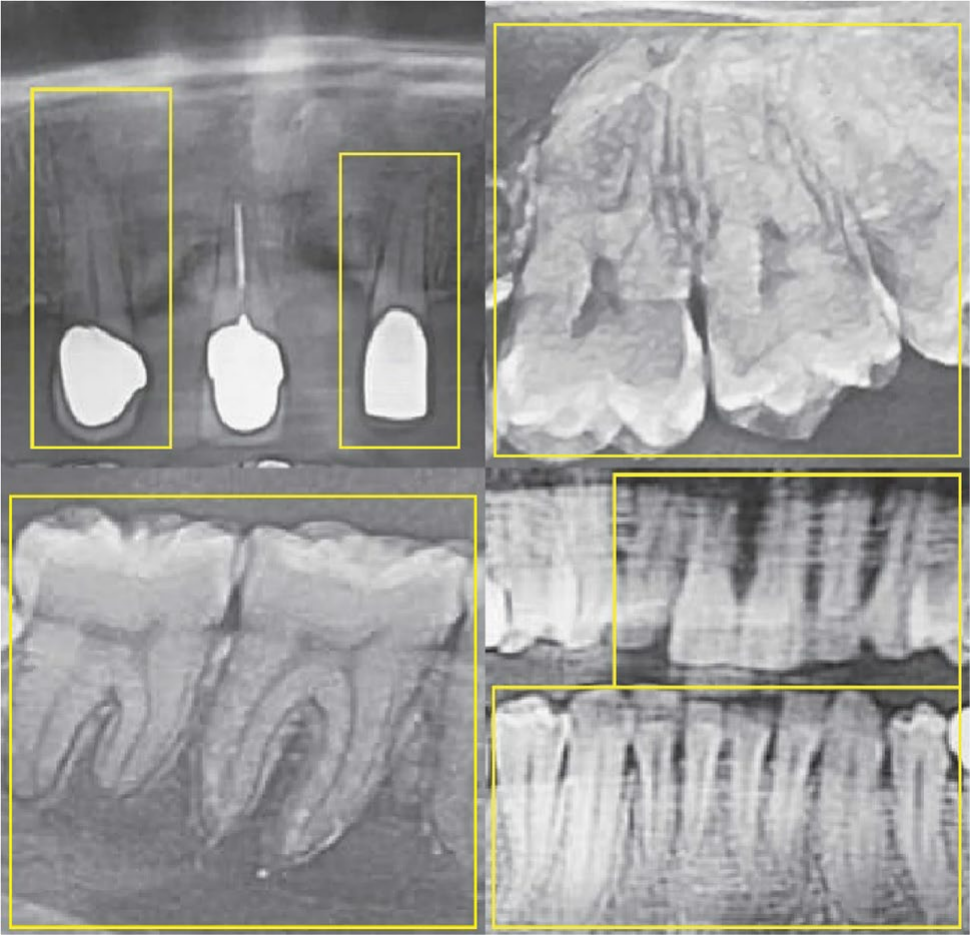
Anatomical and iatrogenic variations within the ‘No endodontic treatment’ class that led to confusing the YOLO models

While the dataset of 240 images in the current study may be deemed small in comparison to larger scale machine learning, deep-learning studies of carious lesions using only 200 radiographs achieved 86% accuracy [28]. The current study can serve as a proof of concept that such models are capable of detecting the condition of canal obturation and can be transferred to larger datasets for more conclusive findings. Several investigations have been proposed to detect carious lesions using convolutional neural networks. It is, however, important to note that some specific classes within the current study, namely ‘No Endodontic Treatment’ and ‘Suboptimal obturation’ were difficult for YOLOv5x to learn. This can be partially attributed to the denoising algorithm over-sharpening images and over-exposing features like bone trabeculae and lamina dura in radiomics of apparently healthy dentition without endodontic treatment. This was when the model began detecting anatomical variables as anomalies. (Fig. 12) As a result, there was a noticeable decline in overall performance for these two classes, with the ‘No Endodontic Treatment’ class experiencing a 25% decrease in accuracy and a very high False Positive Rate of 88%. The imbalance in the dataset in addition caused data skewness and negatively affected the accuracy by 17% of the class ‘total endodontic failure’ that had the smallest amount of data across the classes.

To improve speed of detection, an object detection model was developed to first precisely predict the effects of artifact noise, dataset imbalance, and subsequent augmentation. The results from Tables 1 and 2 show that the underperforming classes serve as a promise that the noisy and imbalanced datasets can further enhance the training accuracy and reduce false positive reports when handled and optimized effectively. As some of the anonymised radiographs were received in physical form, glares and reflections were an evident issue when digitizing the data. All radiographic images therefore were converted to grayscale to improve pixel clarity and decrease color fluctuations. To handle the skewness of the dataset, ‘selective augmentation’ was applied. Past reports identified two successful methods of resampling: under-sampling (removing data from the majority class) and over-sampling (adding repetitive data to the minority class). To account for under-fitting that may result from removing the sample, over-sampling was deemed as the preferred option. However, simply replicating the data would result in over-fitting and therefore data augmentation was performed prior to balancing. The imbalanced dataset was separated into a custom sub-dataset and 3 types of augmentation were applied to prevent biases induced by data duplication. The sub-dataset was reintroduced to the main pool of data, and a 7-level data augmentation was reperformed on the combined dataset. This approach proved to be successful, as seen within the data Tables 2, 3, and 4.

Each image within an object detection task could possess a variety of objects belonging to one or several classifications. Therefore, a model’s classification and localisation had to be examined, where employing accuracy or precision metric alone would be ineffective. Therefore, the final outputs and algorithm performance were evaluated by the mAP metric.

### Limitations and future recommendations

The current system classified stages of possible root canal fillings during endodontic treatment but could not evaluate the amount, for example in millimeters, of over or underfiling present. This can be attributed to the knowledge that deep learning-based object detection models are trained on classes with highly specific class components that have the same visual properties. At present, it is not possible to measure the amount of filling with an object detection model as detection occurs through classifying pixels and bounding boxes and not displacement between objects. An alternative approach to detecting underfilling or overfilling could be achieved when distances are treated as separate classes. However, each class would then require substantial amounts of data and proper labeling for the model to achieve satisfactory results and to avoid data confusion. The difference between underfilling and overfilling property would be highly specific in terms of pixel density as the displacement for the objects are in millimeters. Therefore, a large, clear, high-resolution dataset is required to attain high accuracy which was unavailable in the current study. Finally, YOLOv5x required more CUDA memory to process the data and perform better. Therefore, due to a lack of appropriate hardware infrastructure, and time restraints, more experimentations with YOLOv5x was not considered.

While the practitioners were requested to submit images of obturation performed within the last 1 month, the degree of accuracy of the submitted information was not verified to preserve anonymity and confidentiality. Such an information was not deemed useful in the current study which primarily aimed to teach the model about the different progressive forms of obturation as opposed to the sequalae of relatively stable resolution patterns frequently seen in recall radiographs [29]. Future studies can be carried out to teach the computer-vision model of the different phases of disease resolution following obturation using an elaborate longitudinal dataset.

The following studies can be performed as a continuation of the existing outcomes.

1. Multi-label classification: it is used when there are two or more classes and the data to be classified could belong to none of the classes or all of them at the same time [30]. This issue was experienced when categorizing endodontic treatment outcomes within the current study. The model was frequently seen to misclassify or fail to classify features due to common characteristics of tooth anatomy. Future studies of multi-label classification can aim to sub-divide a category into separate labels and then classify endodontic treatments based on the individual features possessed within the radiomic data.
2. Cost sensitive learning: the ‘no endodontic treatment’ class had average performance in the current study. Therefore, future studies implementing situational sensitive learning may be applied to the said class to identify the costs of prediction error and identify feasibly appropriate augmentation techniques to minimize prediction errors [31].
3. Curriculum learning: a machine-learning technique called curriculum learning is modeled after how people learn by first understanding simpler concepts, and then moving on to information that is more difficult to understand. Previously, Curriculum Learning and its offshoots Self-Paced Learning with Diversity (SPLD) and Self-Paced Learning (SPL) were applied in a number of machine-learning contexts, including Support Vector Machines (SVMs), perceptrons, and multi-layer neural networks, where it was demonstrated that they increased model accuracy and training speed [6]. Transfer learning of the existing model can be applied in a similar manner to extend the classification types of endodontic treatment, provided that there is sufficiently labeled data of cases of under- or overfilling.

## Conclusion

The current study of computer vision applied to radiomic datasets successfully classified endodontic treatment obturation and mishaps according to a custom progressive classification system and serves as a foundation to larger research on the subject matter.

## Data Availability

All data and codes have been provided as supplementary materials with the manuscript. All data and codes have been made available as supplementary information. The computational notebooks can be accessed from an online repository: https://github.com/igenhimel/Endodontic_Treatment_Classification (accessed online on 24 April 2023).

## Footnotes

1 *Class 3 was termed ‘total endodontic failure’ which was revised prior to the submission of the manuscript.*
